# Massive, life-threatening hemoptysis due to localized granulomatosis with polyangiitis

**DOI:** 10.1186/s13019-023-02302-5

**Published:** 2023-07-26

**Authors:** Eleonora Coviello, Francesco Puma, Domenico Pourmolkara, Martina Mandarano, Antonio Giulio Napolitano

**Affiliations:** 1 Department of Thoracic Surgery, University of Perugia Medical School, Piazzale Giorgio Menghini 3, Perugia, 06129 Italy; 2 Thoracic Surgery Unit, Department of Surgical Sciences, Santa Maria della Misericordia Hospital, University of Perugia Medical School, Perugia, 06134 Italy; 3 Department of Anatomic Pathology and Histology, University of Perugia Medical School, Piazzale Giorgio Menghini 3, Perugia, 06129 Italy

**Keywords:** Lobectomy, GPA, Hemoptysis

## Abstract

Massive hemoptysis may be related to a wide spectrum of diseases whose differential diagnosis can be challenging, also due to the medical emergency condition.

We present a case of a 33-year-old woman presented to our department with sudden, life-threatening hemoptysis from unknown etiology, which required a rescue pulmonary lobectomy after resuscitation maneuvers. Histology proved to be a localized Wegener granulomatosis. Our case shows that granulomatosis should always be considered among the possible, although rarer, causes of massive hemoptysis.

## Background

Respiratory infections and malignances are the most frequent causes of hemoptysis in adults [1]. However, only a minority of hemoptysis are life-threatening for the risk of suffocation or, rarely, exsanguination, caused by large amount and rapid bleeding. The various reported diseases, causing massive hemoptysis and respiratory distress, are categorized in Literature in five groups: infective, neoplastic, vascular, autoimmune and drug dependent [3]. Specifically, bronchiectasis, thoracic trauma, lung cancer, tuberculosis, vascular malformation, alveolar hemorrhage and tracheostomy bleeding have been described as the less uncommon causes of massive bleeding originating from the lower respiratory tract [2, 3]. Autoimmune vascular diseases such as Granulomatosis with polyangiitis (GPA), Takayasu, eosinophilic granulomatosis with polyangiitis are included as uncommon cause of hemoptysis but isolated lung clinical manifestations appear definitely rare [4].

The diagnostic work-up has a key role in the identification and consequently in the management of massive hemoptysis, but in the most severe cases the management of the emergency cannot be delayed by any diagnostic protocol [5].

## Case presentation

**Fig. 1:**
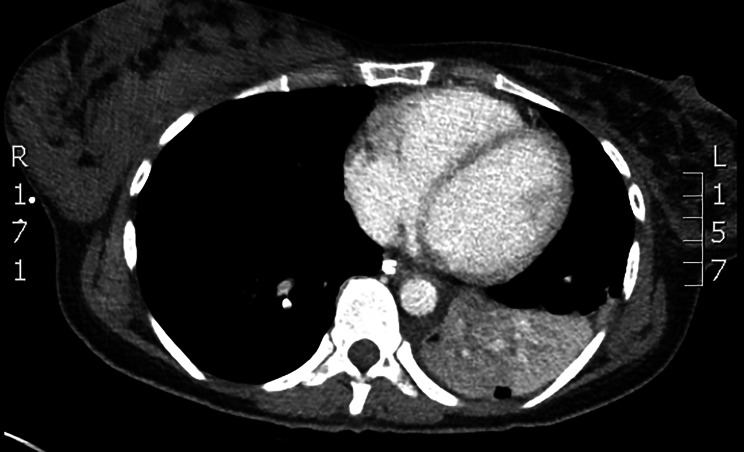
CT scan: complete atelectasis of the left lower lobe due to a blood clot obstructing the lumen; centrolobular nodules and thickened interlobular septa in the left upper lobe; scattered bilateral ground glass opacities

A 33-year-old woman, previously in good condition and breastfeeding for one year, was brought to the emergency department of a neighboring peripheral Hospital because of sudden hemoptysis, associated with asphyxiation and acute arterial hypotension. She underwent immediate single lumen intubation and was transported by ambulance to our Hospital with active bleeding still in progress. A fiberoptic bronchoscopy was performed immediately. The source of bleeding was identified in the left lower lobe bronchus and was controlled by airway toilet, cold saline and adrenaline flushing, intravenous therapy with tranexamic acid and left hemithorax ice packing. Once bleeding stopped, patient underwent a contrast computed tomography (CT) scan which showed complete atelectasis of the left lower lobe due to a blood clot obstructing the lumen; centrolobular nodules and thickened interlobular septa in the left upper lobe; scattered bilateral ground glass opacities; no hypertrophied bronchial arteries (Fig. 1).

A selective bronchial and systemic angiography, followed by pulmonary angiography was then performed, but the certain bleeding source was not identified.

The following day, the patient underwent rigid bronchoscopy. All the blood clots obstructing the left main bronchus and the left lower bronchus were removed, to make evident a reddish vegetating mass obstructing the left lower bronchus. Multiple biopsies were carried out with moderate bleeding. Since the hemostasis was not fully satisfactory, we decided to keep the patient intubated with a 37 Fr double lumen tube.

Roughly thirty hours after rigid bronchoscopy a new episode of massive bleeding occurred (about 300 ml in one hour). The Hemoglobin dropped to 8.9 g/dl. Ventilation was ensured by selective ventilation of the right mainstem bronchus with the double lumen tube and the patient was taken to the operating room in relatively stable respiratory and hemodynamic conditions. Our diagnostic hypothesis was of a possible bleeding carcinoid but indication for surgery was exclusively life-saving. The patient underwent triportal video assisted thoracoscopy (VATS): intraoperative finding demonstrated a complete consolidation of the lower lobe. After dividing the vein with an endoscopic stapler, we found the left lower pulmonary artery branches firmly fixed to the corresponding lobar bronchus. Due to the urgent surgery, conversion to posterolateral thoracotomy was immediately carried out and the left lower lobectomy quickly completed. Finally, mediastinal and hilar lymph nodes sampling was performed. On Day 1, after two blood transfusions, the patient was transferred from the Intensive Care Unit to the ward and discharged on Day 8, after a smooth post-operative course.

**Fig. 2:**
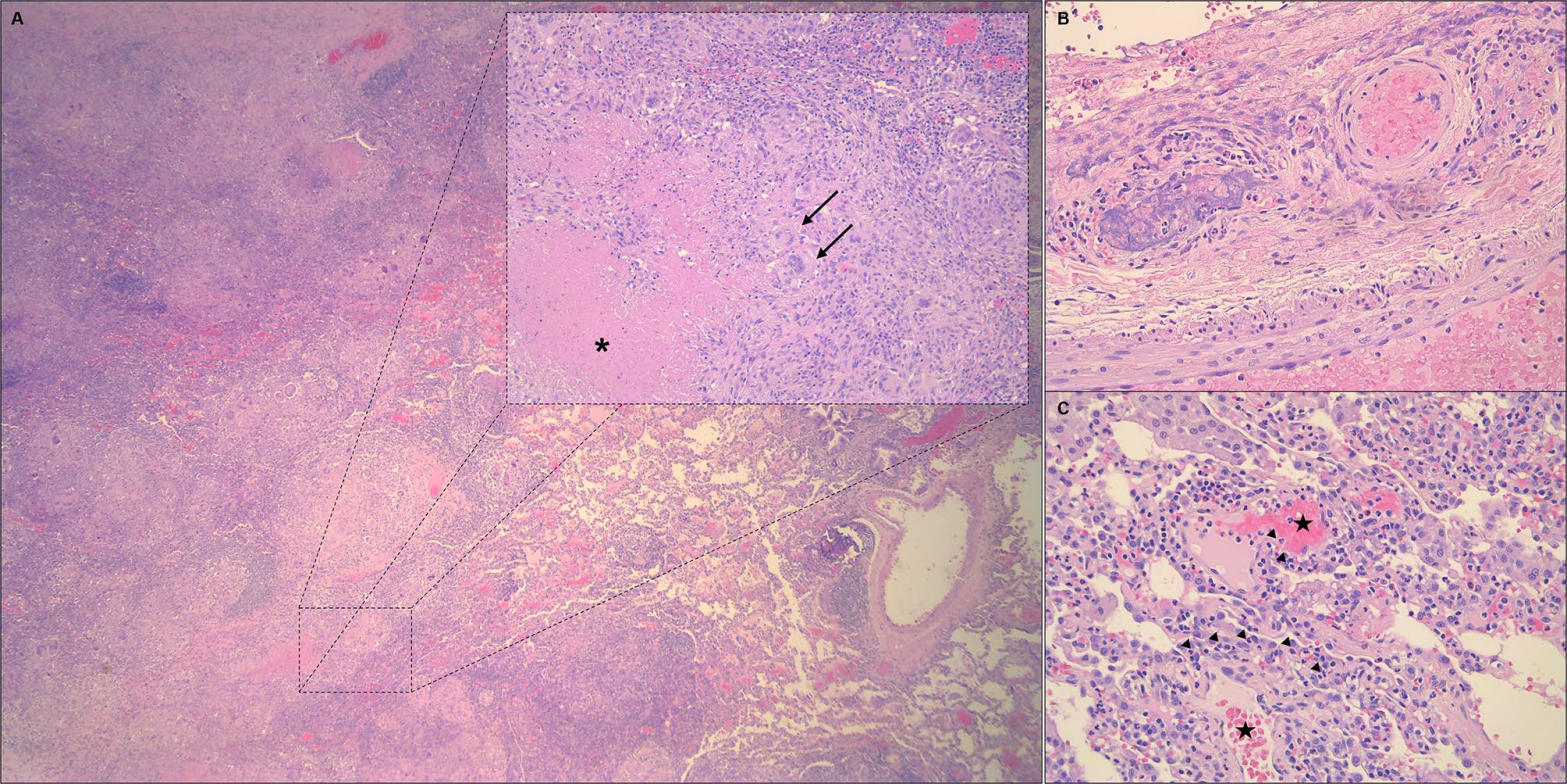
Histopathological findings, Haematoxylin and eosin stain. (**A**) Altered lung parenchima architecture, for the presence of extensive areas of chronic granulomatous, necrotizing (inset, asterisk) inflammation with numerous multinucleated histiocytic giant cells (arrows), in a palisading fashion. (**B**) Detail of necrotizing vasculitis affecting a medium-sized artery. (**C**) Particular of capillaritis, with septal capillaries filled with numerous neutrophils (arrow heads) and focal aspects of alveolar hemorrage (stars). Original magnification: A: 16X; A, inset: 100X; B and C: 200X

The histopathological analysis showed extensive areas of geographic necrosis (Fig. 2A), which subverted the pulmonary parenchyma architecture and which were surrounded by chronic granulomatous inflammation, including numerous multinucleated histiocytic giant cells inflammation (Fig. 2A, inset). Moreover, aspects of necrotizing vasculitis were found, particularly of small- and medium-sized vessels (Fig. 2B), coexisting with septal capillaries filled with inflammatory cells, mainly neutrophils, and aspects of alveolar hemorrhages (Fig. 2C), which are characteristic features of capillaritis. Overall, the histopathological findings were consistent with granulomatosis with polyangiitis.

The patient was finally referred to rheumatologists for the appropriate medical therapy.

## Discussion and conclusions

Granulomatosis with polyangiitis, formerly Wegener’s granulomatosis, is an uncommon immunologically mediated systemic small-vessel vasculitis that is pathologically characterized by an inflammatory reaction pattern that occurs in the upper and lower respiratory tracts and kidneys. It is characterized by necrotizing granulomas of the respiratory tract, vasculitis, and glomerulonephritis. Classically, the acronym ELK (ENT, lungs, kidneys) is used to describe the clinical involvement of the ear, nose and throat, lungs and kidneys [6].

GPA has a wide clinical spectrum ranging from localized disease (predominantly restricted to the respiratory tract) to a severe life-threatening form with involvement of multiple organs (predominantly kidneys and lungs); symptoms can be chronic (in which case they may be misdiagnosed with chronic infections) or acute, involving the respiratory and / or renal system. Diffuse alveolar hemorrhage is a rare potentially lethal complication, which sometimes can be the earliest manifestation of the disease, and can require supportive mechanical ventilation [7–9]. The clinical picture is not pathognomonic due to the presence of cough, hemoptysis and progressive dyspnea. The most common chest CT findings are also absolutely nonspecific [10, 11]: pulmonary nodules including unilateral nodules, solitary nodule, ground-glass nodules, cavitary nodules, mass with or without cavitations, pulmonary consolidations can be misdiagnosed with infections, post obstructive pneumonia, abscesses, tumors and interstitial lung abnormalities [12–14].

Achieving differential diagnosis between vasculitis can be very tricky. Usually, combination of clinical, radiological and laboratory findings (positive Antineutrophil Cytoplasmic Antibodies (ANCA) serology), and histology (evidence of necrotizing vasculitis) leads to the final diagnosis.

Our patient presented with massive hemoptysis associated with anemia and respiratory distress, emergency treated with endotracheal intubation for airway control. Flexible bronchoscopy was performed showing a supposed endobronchial lesion obstructing left lower bronchus. Chest CT scan was nonspecific and demonstrated only complete atelectasis of the left inferior lobe due to blood clot obstructing the lumen, centrolobular nodules and thickened interlobular septa in the left upper lobe and scattered bilateral ground glass opacities, related to the recent bleeding. Except from anemia and minimal increased white cells blood count, no other laboratory findings were present, even though the ANCA serology was not tested; no significant anamnestic data and no comorbidities were reported. A bleeding endobronchial tumor was our first diagnostic hypothesis considering the patient’s age, chest CT scan, and bronchoscopic findings, but the severity of clinical onset did not allow for further investigation or waiting for biopsy results [15, 16].

Literature review counts of several cases of GPA with diffuse hemorrhagic alveolar manifestation, but only a few with severe hemoptysis [12–14]. In particular, Our case appears to be the first GPA with so massive, life-threatening hemoptysis that required intubation to airways control and surgical treatment as unique management, without time to elaborate the diagnosis.

ANCA serology probably would have led to the diagnosis but was not performed because GPA was wrongly not considered as the possible cause of such severe hemoptysis. However, the correct diagnosis would not have avoided the rescue surgery after the second massive episode of bleeding.

## Data Availability

Not applicable.
